# Seroprevalence of Hepatitis B and C among Children in Endemic Areas of Turkey

**Published:** 2010-03-01

**Authors:** Murat Kangin, Mine Turhanoglu, Serda Gulsun, Bahri Cakabay

**Affiliations:** 1 Department of Pediatrics, Diyarbakir Education and Research Hospital, Diyarbakir, Turkey; 2 Department of Microbiology, Diyarbakir Education and Research Hospital, Diyarbakir, Turkey; 3 Department of Infectious Diseases and Clinical Microbiology, Diyarbakir Education and Research Hospital, Diyarbakir, Turkey; 4 Department of General Surgery, Diyarbakir Education and Research Hospital, Diyarbakir, Turkey

**Keywords:** Hepatitis B Virus, Hepatitis C Virus, Hepatitis Markers, Children, Seroprevalence

## Abstract

**Background and Aims:**

Hepatitis B virus (HBV) and Hepatitis C virus (HCV) infections are major worldwide public health problems. The objectives of this study were to evaluate the seroprevalence and epidemiological profile of hepatitis B and hepatitis C, to determine the impact of the national vaccination programme against hepatitis B on the prevalence of the hepatitis B surface antigen (HBsAg) carrier and the antibody to hepatitis B surface antigen (anti-HBs) occurrence rate among 0-14 year-old children in southeast Turkey.

**Methods:**

The seroprevalence of hepatitis B and hepatitis C markers was evaluated retrospectively in a group of 10,391 children who were admitted to a tertiary hospital, the Diyarbakir Education and Research Hospital, from January 2005 to December 2008, in order to obtain a better understanding of the regional hepatitis seroprevalence. Children were divided into three different age groups: pre-education period (0-6 years), primary school period (7-12 years) and secondary school period (13-14 years). Samples were analyzed for HBsAg, hepatitis B e antigen (HBeAg), antibody to HBeAg (anti-HBe), anti-HBs positive/antibodies to hepatitis B core antigen (anti-HBc) positive, isolated anti-HBs and antibodies to Hepatitis C virus (anti-HCV) using a commercially available enzyme-linked immunosorbent assay (ELISA).

**Results:**

The mean age of all participants was 8.5± 2 years (range, 0-14). The overall percentages for the prevalence of HBsAg, HBeAg, anti-HBe and anti-HCV were 8.1%, 2.1%, 5.9% and 0.5%, respectively. HBsAg seroprevalence differed significantly by age and gender (P < 0.001). HBeAg seroprevalence was high in the earliest years (P < 0.01). The overall prevalence of anti-HCV did not differ significantly by age (P > 0.5) but differed by gender (P < 0.001). The overall percentages for the prevalence of isolated anti-HBs and anti-HBs positive/anti-HBc positive were 34.2% and 56.9%,respectively.

**Conclusions:**

Our study sheds new light on hepatitis seroprevalence in southeastern Turkey. For example, 1) The seroprevalence of hepatitis B in southeast Turkey is still at its highest rate, according to the averages reported in other studies conducted in the same and different regions of Turkey; and it has not decreased, as reported previously. 2) HBeAg seroprevalence in the earliest years of childhood is high in our study; this is evidence for early acquisition of the infection.3) Isolated anti-HBs positive and anti-HBs positive/anti-HBc positive prevalence is high; given these features, it is obvious that despite the high incidence of vaccinated children, the prevalence of hepatitis B is increasing; and children acquire these viruses in their earliest years. 4) We found the overall prevalence of HCV infection unchanged. Our region has a low endemicity for HCV.

## Introduction

It is estimated that 2 billion people are infected with hepatitis B virus (HBV) worldwide, and 350 million of them are chronic carriers [1].

The prevalence of HBV infection has been reported to be different in various parts of the developed and developing world [1]. Turkey has intermediate endemicity for HBV [2]. The hepatitis B surface antigen (HBsAg) carrier rate is 4% (2-8%), while antibody to hepatitis B surface antigen (anti-HBs) positivity is 30% (20-55%). On average, 40%(30-70%) of patients with chronic liver disease are HBsAg positive.The prevalence of HBV infection and the predominant mode of transmission vary greatly depending on the geographical region and epidemiologic factors in Turkey [2][3]. These figures are lower in Western Turkey, and much higher in East and South-east Turkey where socio-economic levels are lower [2].

Prevention of childhood HBV infection has a large impact on the prevalence of chronic HBV infection and its sequelae [4]. In Turkey, the reporting of acute viral hepatitis is compulsory and hepatitis B vaccine is given at birth to children whose mothers are known to be HBsAg positive. Our country has added HBV vaccine to its routine childhood immunization program and has provided it to all children since 1997.

There are insufficient data on the seroepidemiology of HBV infection in children living in southeast Turkey. In this retrospective study we aimed at determining the actual prevalence of hepatitis B and hepatitis C and also the impact of the national vaccination program on hepatitis B infection in our region, by observing the records of 10,391 children, over a period of 4 years from January 2005 to December 2008.

## Materials And Methods

### Study settings and patients

Diyarbakir is the largest city in southeastern Turkey. Situated on the banks of the River Tigris, it is the administrative capital of Diyarbakir province, with a population of almost 1.5 million [5]. The Diyarbakir Education and Research Hospital is the only and the biggest tertiary state hospital serving the region. Hepatitis B seroprevalence is at its highest rate in southeast Turkey, compared to other regions of Turkey. Our study, which covered the period January 2005-December 2008, was carried out at the Diyarbakir Education and Research Hospital, and included children without icterus. In addition, none of the children had a history of blood transfusion. Informed consent was obtained from the children's parents and/or relatives and this study was approved by the local health directorate and the ethics committee of the Diyarbakir Education and Research Hospital. The records of a total of 10,391 applicants were analyzed retrospectively in order to determine the seroprevalence of hepatitis B and hepatitis C; also the impact of a routine active vaccination program on individuals in this endemic region was investigated.

### Serological testing

The serological markers of HBsAg, hepatitis B e antigen (HBeAg), antibody to HBeAg (anti-HBe), antibody to hepatitis B core antigen (anti-HBc), anti-HBs and antibody to hepatitis C virus (anti-HCV) were investigated retrospectively in serum samples sent from policlinics and clinics to the microbiology laboratory. In the laboratory, commercial kits based on the enzyme-linked immunosorbent assay (ELISA) E170 (Roche, Germany) method were used. Positive samples were subsequently tested for hepatitis B virus DNA (HBV-DNA) and hepatitis C virus RNA (HCV-RNA) at Hifzisihha National Public Laboratories using the polymerase chain reaction (PCR) technique. In endemic regions of Turkey, vertical transmission is an important route, so we selected the following age groups: pre-education period (0-6 years), primary school period (7-12 years) and secondary school period (13-14 years).

### Statistics

Statistical analysis was carried out by using the SPSS 10.0 program. The chi-square test was used in the statistical analyses. ANOVA was used for analyzing multiple groups. A value of P < 0.05 was considered statistically significant.

## Results

Retrospectively, applicants totaling 10,391, with an average age of 8.5± 2 years (5,998 males (mean age 9.3±2 years) and 4,393 females (mean age 7.7±2 years) were tested for hepatitis B and C markers from January 2005 to December 2008 Table 1. In this study, we aimed at determining the seroprevalence of hepatitis B and hepatitis C, as fwell as the frequency of HBe/anti-HBe, anti-HBs positive/anti-HBc positive and isolated anti-HBs positivity. Our aim was also to compare vaccination and environmental health care program results among children 0-14 years over a period of 4 years.

**Figure 1:**
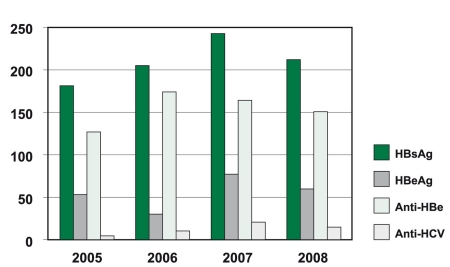
Seroprevalence of HbsAg, Hbe-Ag, Anti-HBe and anti-HCV

The overall percentages for the prevalence of HBsAg, HBeAg, anti-HBe and anti-HCV were 8.1%, 2.1%, 5.9% and 0.5%, respectively. As seen in Fig.1, the fprevalence of HBsAg and HBeAg has not decreased in the region despite the implementation of active hepatitis B vaccine programs and environmental health policies by the Ministry of Health.

**Table 1:** Hepatitis seroprevalence by sex and age.

**Age Group**	**Sex (Female/Male)**	**Number of tested**	**HBsAg**	**HBeAg**	**Anti-HBe**	**Anti-HCV**	**Isolated anti-HBs**	**Anti-HBs positive/Anti-HBc positive**
**0-6**	1260/1901	3721	278	85	193	23	402	961
**7-12**	1745/2275	3470	423	107	316	12	1301	2655
**13-14**	1388/1822	3200	141	30	111	18	1857	2300
**Total**	4393/5998	10391	842	222	620	53	3560	5916

**Figure 2:**
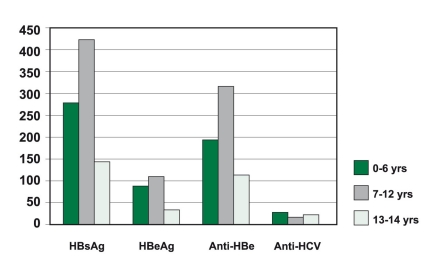
Hepatitis seroprevalence by age.

HBeAg seroprevalence was 2.1% overall with a considerable increase in children aged =12 years (P < 0.01) Fig. 2 but did not differ by gender (P > 0.05). This shows evidence for early acquisition of the infection. We found that the overall prevalence of HBsAg differs significantly by gender and age (P < 0.001). A higher prevalence of HBsAg was found in the 7-12 year-old group and in the male gender (P < 0.001).

**Figure 3:**
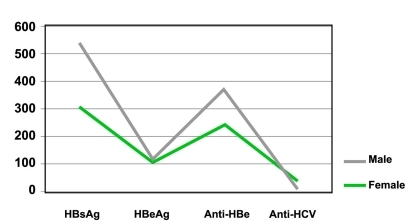
Hepatitis seroprevalence by gender.

Fifty three (mean age: 10.1±2 years) cases, in toto, were confirmed to be infected with HCV. The seroprevalence of anti-HCV among healthy people was 0.5%. The overall prevalence of anti-HCV did not differ significantly by age (P > 0.05), but differed by gender (P < 0.001). The prevalence of HCV infection was more frequently seen in females than in males (36 females vs. 17 males). In contrast, HBV infection was found to be more common in males than in females (63.8% vs. 32%) Fig. 3. Furthermore, the mean age of subjects with HBV infection was significantly lower than that of subjects with HCV infection (8.5±2 years vs. 10.1±2 years, P < 0.001).

**Figure 4:**
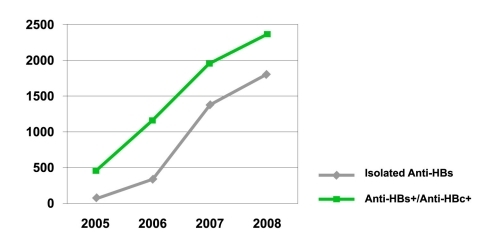
Seroprevalence of Isolated anti-HBs and anti-HBs positive/anti-HBc positive.

**Figure 5:**
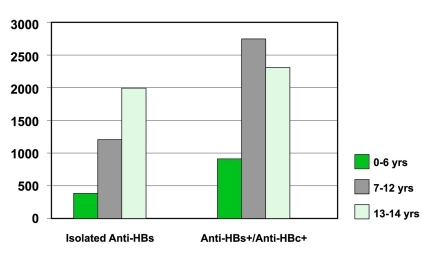
Isolated anti-HBs and anti-HBs positive/anti-HBc positive by age.

The overall percentages for the prevalence of isolated anti-HBs and anti-HBs positive/anti-HBc positive were 34.2% and 56.9%, respectively. In the statistical analyses, anti-HBs positive/anti-HBc positive and anti-HBs differed significantly by age (P < 0.01). As seen in Fig. 4 and 5, isolated anti-HBs positive and anti-HBs positive/anti-HBc positive prevalence was high; taking this into account, it is obvious that despite the high incidence of vaccinated children, the prevalence of hepatitis B is increasing, and children acquire these viruses in their early years.

## Discussion

The prevalence of viral hepatitis and the outcomes of immunization programs among 0-14 year-olds have not been extensively studied so far in our region. The objective of this study was to determine the exact seroprevalence of HBV and HCV infections in 0-14 year-old children living in the endemic region of Turkey, as well as to correlate the serological results with the epidemiological data.Turkey is in a middle-endemic region (2-8%) for hepatitis B. According to the National Study Group, the national HBsAg positivity rate was found to be 6.2% in our country, for children between 6 and 10 years of age [6]. The east and southeast region of Turkey is known as an endemic area for HBV infection in Turkey [7]. We found the seroprevalence rate of HBsAg to be 8.1%. Previous studies performed in the same region revealed HBsAg a seroprevalence rate of between 9.5 and 11.5% [8][9]. Ucar et al. found HBV positivity of 4.6% and 5.2% in children aged 6-8 and 9-12 years, respectively [10].Degertekin investigated HBV among schoolchildren in the same region and found HBsAg positivity of 2%, 5% and 7.5% in the 7, 12 and 17 year-old groups, respectively [11]. In studies performed in southeast Turkey, the high prevalence of hepatitis B has been explained by low living conditions, low-level socioeconomic conditions and a high rate of vertical and horizontal transmission [7]. In some of the previous studies, a low HBsAg prevalence of 4-6% has been found for our region, probably due to small sample sizes and to heterogeneous case properties such as living in rural areas or among schoolchildren.

Turkey is a non-endemic area for HCV infection [7]. According to different studies conducted in our country, it has been reported that anti-HCV prevalence varies between 0.3 and 1.8% in blood donors [12]. Prevalence studies conducted in our region found anti-HCV in similar ratios. Dursun et al. found an HCV prevalence of 0.4%, whereas Dikici et al. found a rate of 0.56% in our region [13][14]. Emekdas et al. found the seroprevalence of HBV and HCV to be 4.19% and 0.38%, respectively [7].

In the pediatrics population, among 6 to 10-year-olds, the Turkish National Research Group found the anti-HBs ratio to be 12.1% [6]. Demirturk et al. found isolated anti-HBs and isolated immunoglobulin G antibody to HBc (anti-HBc IgG) to be 27.2% and 6.1% in mid-central Anatolia [15]. Araz found anti-HBs positivity to be 41.7% in a pediatric group admitted to a pediatric hospital located in southeast Turkey [16]. Isolated anti-HBs positive and anti-HBs positive/anti-HBc positive prevalence is high in our study (34.2% and 56.9%). Taking all this into account, it is obvious that despite the high incidence of vaccinated children, there is an increasing number of self-immune children, and generally children acquire these viruses in their first few years of life. Our observation of a gradual increase in the seroprevalence of anti-HBs with age is in fact an indicator of effective national routine vaccination against hepatitis B. Thus, it is obvious that the routine active vaccination program is effective in our region; but in spite of this vaccination program and the other active environmental health policies now being implemented in the region, hepatitis B continues to rise.

Most of the seroepidemiological studies from Turkey have shown that horizontal transmission was the major mode of transmission [2][11][17]. Our observation of a high HBsAg and HBeAg prevalence in the first few years of life, and the high prevalence of anti-HBs positive/anti-HBc positive reveals that vertical transmission plays an important role in the transmission of HBV in our region.

In Van, another big city in the region, Turkdogan et al. [18] found HBsAg and anti-HBs seropositivity as 9.5% and 44.4%, respectively. In the same region, Uner et al. [19] found HBs Ag seropositivity was 9.85%. In their study they emphasized the importance of vertical transmission of HBV infection in the perinatal period, and a lesser possibility of intrafamilial spread of the disease in the region.

Data originating from HBV-screening studies of children are not satisfactory in Turkey. In most studies, HBsAg is the only parameter investigated. There have been few studies comparing the prevalence of other hepatitis markers in our region.

Seroepidemiological studies of hepatitis are necessary, especially in endemic regions, because these studies not only show us the regional and geographical epidemiology of the disease, but also help us to develop regional preventive strategies. Our findings suggest that the incidence of hepatitis B infection is not decreasing in southeast Turkey, and our study reveals that the seroprevalence of HBsAg is higher than the averages reported in other studies conducted not only in the same but also in different regions of Turkey. Significantly high prevalence of HBsAg and HBeAg positivity among children younger than 12 years of age is definite evidence for early acquisition of the infection (P < 0.001). In our region, nearly everyone is infected during childhood. Major probable causes may be due to the low quality of life especially in rural areas, , children living in crowded households, the uncontrolled movement of populations and the high birth rate in the region.

## Conclusions

Consequently, we have shown that HBV is still endemic in southeast Turkey and it has not decreased, as had been reported previously. Also, higher HBeAg positivity rates show evidence for early acquisition of the infection and supports the fact that in addition to a horizontal transmission route, vertical transmission plays an important role in the transmission of hepatitis in our region. In addition, this study has emphasized that a routine hepatitis immunization program is effective, but that targeting prophylaxis has failed to control HBV infection. Thus additional measures are needed. We suggest an immediate intervention strategy to prevent the spread of HBV infection. HBV vaccination should be continued and HBV serological tests in pregnant women must be obligatory to avoid viral vertical transmission. Educational and awareness activities are necessary, and it is urgent that immediate effective birth control measures, due to the high birth rate in the region, be taken. Furthermore, studies like this one are needed for further knowledge about our success in the fight against hepatitis.
